# Transcriptional expressions of Chromobox 1/2/3/6/8 as independent indicators for survivals in hepatocellular carcinoma patients

**DOI:** 10.18632/aging.101658

**Published:** 2018-11-27

**Authors:** Gang Ning, Yan-Lin Huang, Li-Min Zhen, Wen-Xiong Xu, Qian Jiao, Fang-Ji Yang, Li-Na Wu, Yong-Yuan Zheng, Jie Song, Yen-Sheng Wang, Chan Xie, Liang Peng

**Affiliations:** 1 Department of Infectious Diseases, the Third Affiliated Hospital of Sun Yat-Sen University, Guangzhou, China; 2 Guangdong Provincial Key Laboratory of Liver Disease Research,the Third Affiliated Hospital of Sun Yat-sen University, Guangzhou, China; 3 Department of Environmental Health Science, Yale School of Public Health, New Haven, Connecticut 06520, USA; * Equal contribution

**Keywords:** hepatocellular carcinoma, CBX, prognosis, ONCOMINE, Kaplan-Meier plotter

## Abstract

Chromobox (CBX) proteins are important components of epigenetic regulation complexes known to play key roles in hepatocellular carcinoma (HCC). Little is known about the function of distinct CBXs in HCC. To address this issue, the study investigated the roles of CBXs in the prognosis of HCC using ONCOMINE, UALCAN, Human Protein Atlas, Kaplan-Meier Plotter, *c-BioPortal* databases. Over expressions of 8 CBXs members were found to be significantly associated with clinical cancer stages and pathological tumor grades in HCC patients. Besides, higher mRNA expressions of CBX1/2/3/6/8 were found to be significantly associated with shorter overall survival (OS) in HCC patients, while higher mRNA expression of CBX7 was associated with favorable OS. Multivariate analysis also showed that high mRNA expressions of CBX1/2/3/6/8 were independent prognostic factors for shorter OS of HCC patients. Moreover, high mutation rate of CBXs (51%) was also observed in HCC patients, and genetic alteration in CBXs was associated with shorter OS and disease-free survival (DFS) in HCC patients. Taken together, these results indicated that CBX1/2/3/6/8 could be prognostic biomarkers for survivals of HCC patients.

## Introduction

Hepatocellular carcinoma (HCC) represents 90% of the primary liver cancer and is the fifth leading cancer that ranked second in cancer-related death worldwide. The incidence has increased rapidly over the years and the mortality rate is unfavorable. Studies suggested patients with HCC could not survive by more than 5 years [1,2]. Efforts have been made in studying the mechanisms of the development, progression and metastasis of HCC; however, the molecular characteristics of HCC, to date, remain unknown. By understanding the underlying pathogenesis and etiology of HCC would show light in discovering the advanced treatment and diagnostic biomarkers.

Certain cancer genetics such as mutations, small nucleotide polymorphism (SNP), translocations, deletions, and insertions could contribute to the genetic regulation of cancers [3]. Moreover, recent studies suggested aberration of epigenetic regulation also played pivotal roles in HCC regulations [4]. Polycomb group (PcG) complexes are epigenetic regulatory complexes, dysregulation of which has been associated with many cancer types [5–7]. Chromobox (CBX) family proteins are canonical components of PcG that regulate tumorigenesis and progression of many cancers including HCC by inhibition of cell differentiation and self-renewal of cancer stem cells [8,9]. A comprehensive study of distinct CBXs family members in HCC will help to uncover the molecular mechanisms involved in the development of HCC and could unveil novel prognostic and therapeutic targets for the devastating disease.

To date, 8 CBXs family proteins have been identified in human genomes. All of them take part in the regulation of heterochromatin, gene expression, and developmental programs. Based on the molecular structure of CBXs family proteins, they can be subdivided into two groups: HP1 group (includes CBX1, CBX3 and CBX5) and Pc group (includes CBX2, CBX4, CBX6, CBX7, and CBX8). HP1 group consists of an N-terminal chromodomain and a C-terminal chromoshadow domain, while Pc group contains only a conserved N-terminal chromodomain [10]. It is worth noting that functions of different CBXs family proteins are correlated with distinct regions of chromatin and are non-overlapped in embryonic stem cells [11–13].

Previous studies have found aberrant expressions and their prognostic values in some members of CBXs family. For instance, CBX4 was over-expressed in clinical tissues and multiple HCC cell lines. High expression of CBX4 was associated with tumor size, pathologic differentiation and poorer survival of patients, while down-regulation of CBX7 was found to be associated with shorter overall survival (OS) of HCC patients [14,15]. Nevertheless, the role of distinct CBXs family members remained unknown in the development and progression of HCC. In the present study, we addressed this problem by analyzing the expression and mutations of different CBXs family members and their relations with clinical parameters in HCC patients. Furthermore, we also analyzed the predicted functions and pathways of the mutations in CBXs as well as their 50 frequently altered neighbor genes.

## RESULTS

### Over-expression of different CBXs family members in patients with HCC

**Figure 1:**
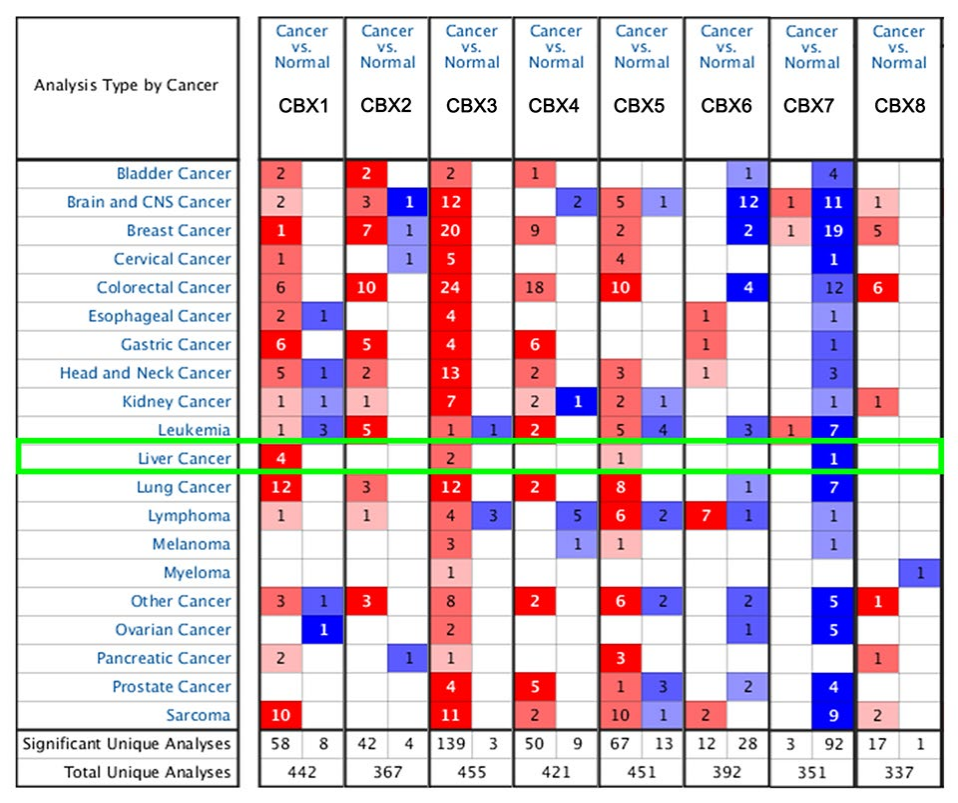
**Transcriptional expression of CBXs in 20 different types of cancer diseases (ONCOMINE database).** Difference of transcriptional expression was compared by students’ t-test. Cut-off of *p* value and fold change were as following: *p* value: 0.01, fold change: 1.5, gene rank: 10%, data type: mRNA.

**Figure 2:**
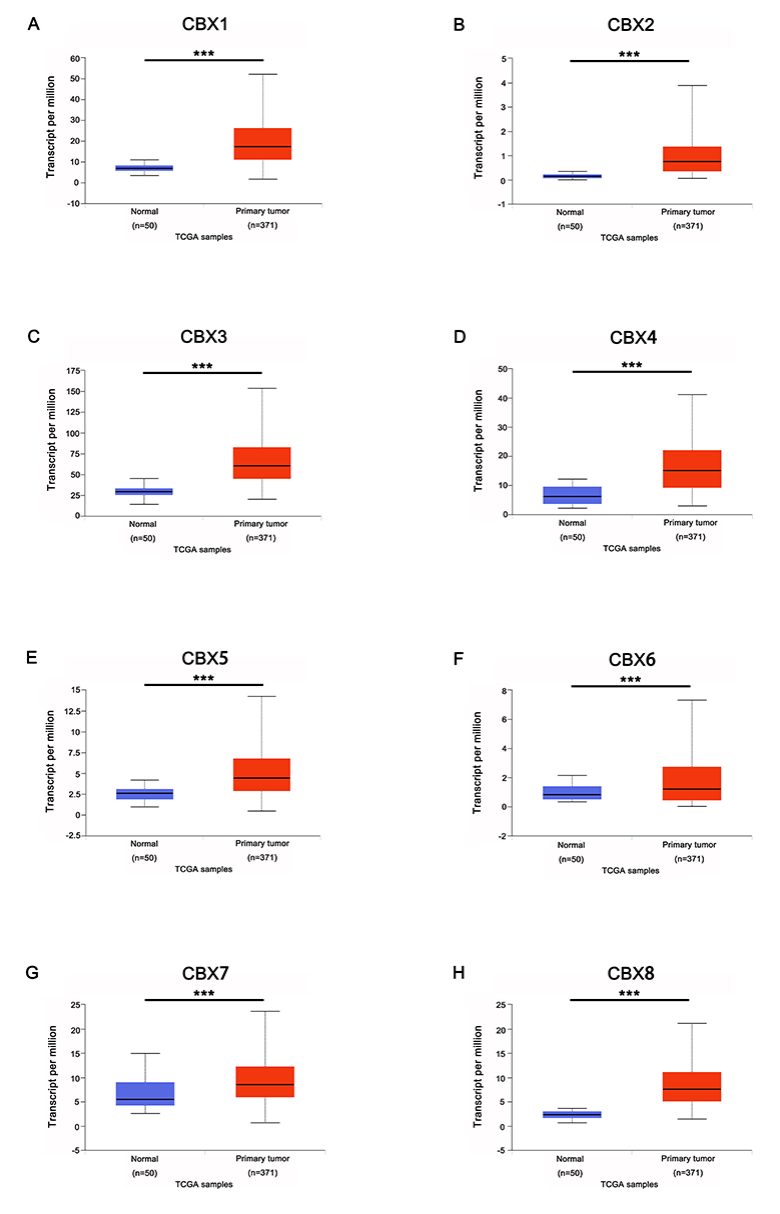
**mRNA expression of distinct CBXs family members in HCC tissues and adjacent normal liver tissues (UALCAN*).*** mRNA expressions of 8 CBXs family members were found to be over-expressed in primary HCC tissues compared to normal samples (**A-H**). *** p<0.001.

In order to explore the distinct prognostic and potential therapeutic value of different CBXs members in HCC patients, mRNA expression and protein expression were analyzed by ONCOMINE database (www.oncomine.org), UALCAN (http://ualcan.path.uab.edu), and Human Protein Atlas (https://www.proteinatlas.org). As were shown in Figure 1 and Table 1, mRNA expressions of 8 CBXs family members in 20 types of cancers were first measured and compared to normal tissues by ONCOMINE database. Significantly higher mRNA expressions of CBX1/3/5 were found in HCC tissues in multiple datasets. In Roessler Liver 2 dataset, CBX1 over-expression was found in HCC tissues compared with normal tissues with a fold change of 2.688 (p=4.33E-80) [16], while Wurmbach observed 1.781-fold increase in CBX1 mRNA expression in HCC samples (p=4.38E-8) [17] and Roessler found 2.405-fold increase in CBX1 mRNA expression in HCC tissues (p=1.37E-7) [16]. Significant up-regulation of CBX3 was also found in HCC tissues compared to normal tissues. The result from Roessler dataset showed that there were 1.888-fold (p=7.91E-63) and 1.704 fold (p=2.12E-5) increase in CBX3 mRNA expression in HCC tissues, respectively [16]. Similarly, in Roessler Liver dataset, 1.662-fold increase in CBX5 mRNA expression was found in HCC tissues compared to normal tissues (p=7.47E-6) [16]. Next, the mRNA expression patterns of 8 CBXs family members were further measured by UALCAN whose resources were based on level 3 RNA-seq and clinical data from 31 cancer types of TCGA database, which was different from ONCOMINE database. As was shown in Figure 2, mRNA expressions of 8 CBXs members were all found to be significantly up-regulated in primary HCC tissues compared to normal samples (all p<0.05).

**Table 1:** Significant changes of CBXs expression in transcription level between HCC and normal liver tissues (ONCOMINE).

	**Types of HCC VS. Liver**	**Fold Change**	**P value**	**t-test**	**Ref**
CBX1					
	Hepatocellular Carcinoma	2.688	4.33E-80	23.586	Roessler Liver 2 [25]
	Hepatocellular Carcinoma	1.781	4.38E-08	6.462	Wurmbach Liver [26]
	Hepatocellular Carcinoma	2.405	1.37E-07	6.649	Roessler Liver [25]
CBX3					
	Hepatocellular Carcinoma	1.888	7.91E-63	19.952	Roessler Liver 2 [25]
	Hepatocellular Carcinoma	1.704	2.12E-05	4.71	Roessler Liver [25]
CBX5					
	Hepatocellular Carcinoma	1.662	7.47E-06	5.161	Roessler Liver [25]
					

HCC: hepatocellular carcinoma; CBX: chromobox

**Figure 3:**
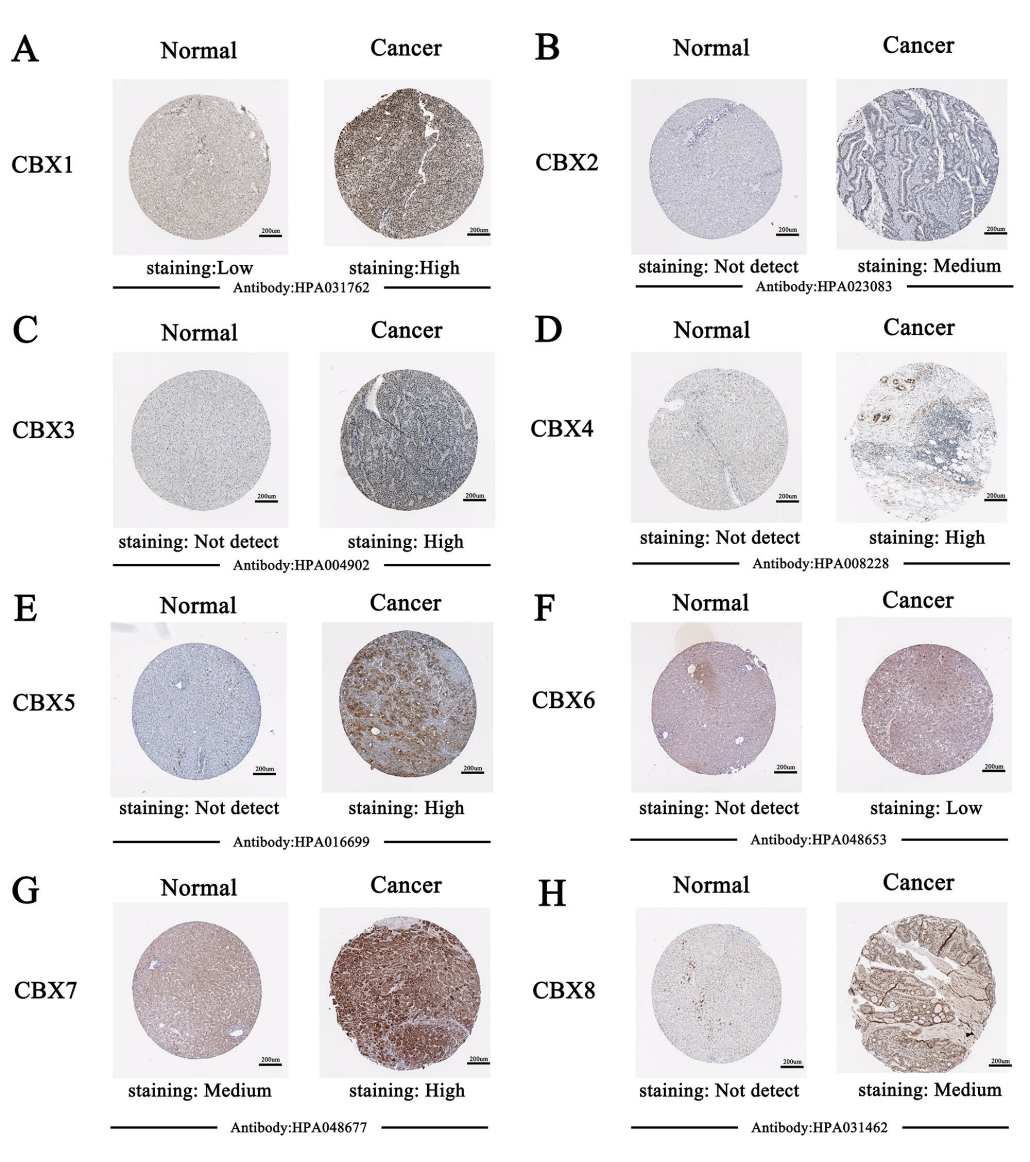
**Representative immunohistochemistry images of distinct CBXs family members in HCC tissues and normal liver tissues (Human Protein Atlas*).*** CBX2/5/7/8 proteins were not expressed in normal liver tissues, whereas their low and medium expressions were observed in HCC tissues (**B, E, G-H**). Low protein expressions of CBX1/3/4 were found in normal liver tissues, while their medium and high protein expressions were observed in HCC tissues (**A, C-D**). Low protein expression of CBX6 was observed both at normal liver tissues and HCC tissues (**F**).

After examining the mRNA expression patterns of CBXs in HCC, we tried to explore the protein expression patterns of CBXs in HCC by the Human Protein Atlas. As was shown in Figure 3, CBX2/5/7/8 proteins were not expressed in normal liver tissues, whereas low and medium expressions of them were observed in HCC tissues (Figure 3B, E, G-H). In addition, low protein expressions of CBX1/3/4 were expressed in normal liver tissues, while medium and high protein expressions of them were observed in HCC tissues (Figure 3A, C-D). However, low protein expression of CBX6 was observed both at normal liver tissues and HCC tissues (Figure 3F). Taken together, our results showed that transcriptional and proteinic expressions of CBXs were over-expressed in patients with HCC.

### Association of mRNA expression of different CBXs family members with clinicopathological parameters of HCC patients

**Figure 4:**
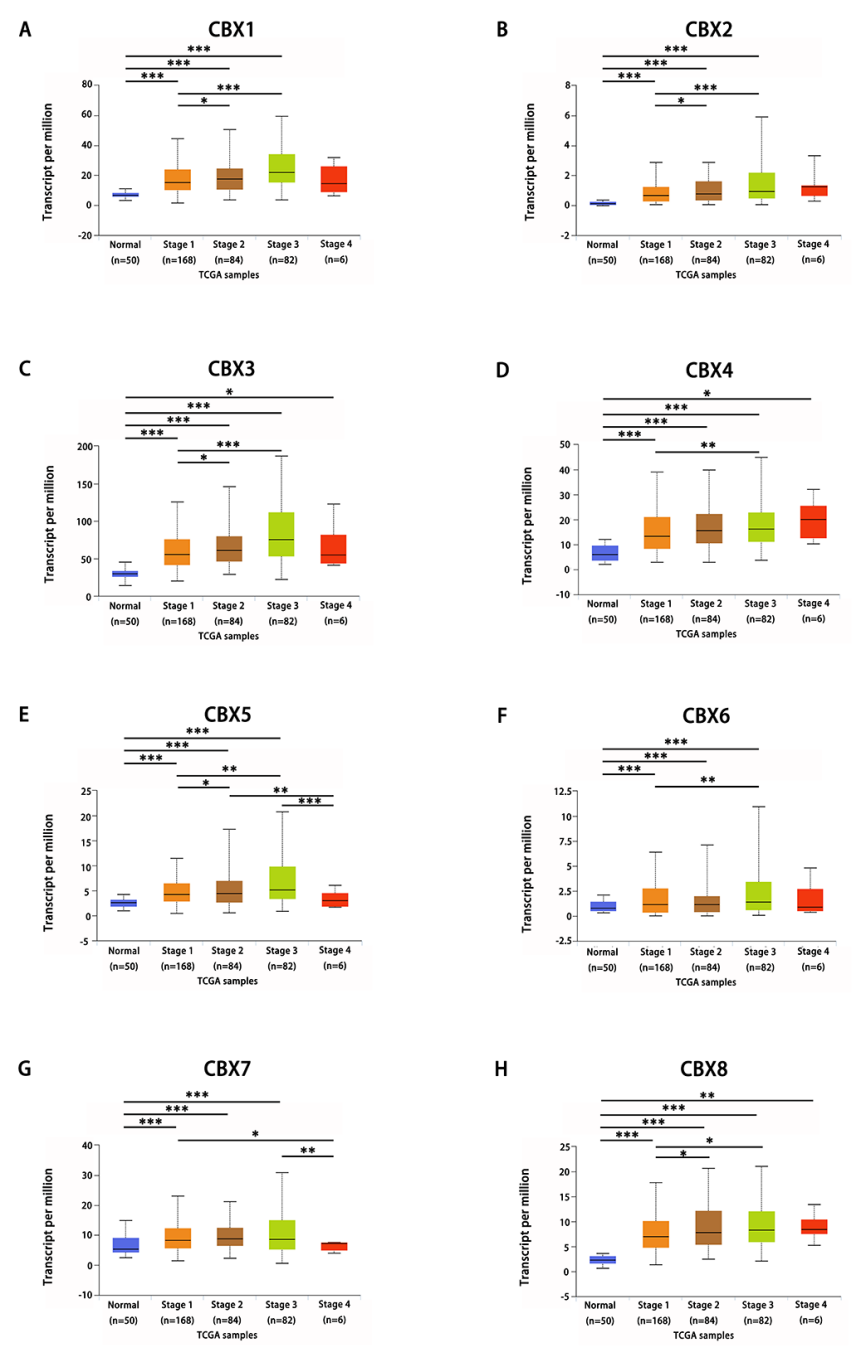
**Relationship between mRNA expression of distinct CBXs family members and individual cancer stages of HCC patients.** mRNA expressions of 8 CBXs family members were remarkably correlated with patients’ individual cancer stages, patients who were in more advanced stages tended to express higher mRNA expression of CBXs. The highest mRNA expressions of CBX4/8 were found in stage 4 (**D, H**), while the highest mRNA expressions of CBX1/2/3/5/6/7 were found in stage 3 (**A-C, E-G**). *p<0.05, **p<0.01, ***p<0.001.

**Figure 5:**
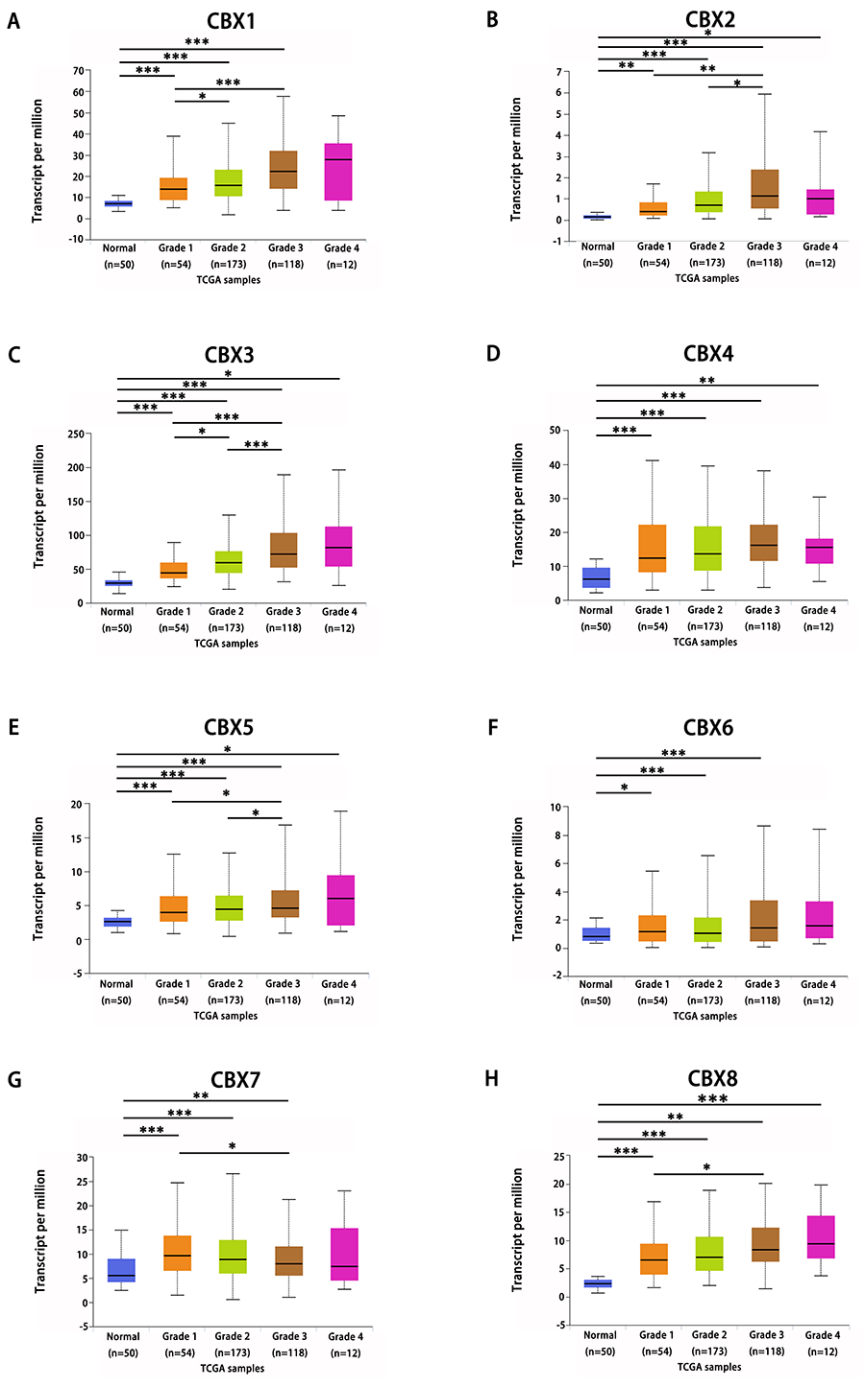
**Association of mRNA expression of distinct CBXs family members with tumor grades of HCC patients.** mRNA expressions of 8 CBXs family members were significantly related to tumor grades, and as tumor grade increased, the mRAN expressions of CBXs tended to be higher. The highest mRNA expressions of CBX1/3/4/5/6/8 were found in tumor grade 4 (**A, C-F, H**), while the highest mRNA expression of CBX2 was found in grade 3 (**B**). However, the highest mRNA expression of CBX7 was found in grade 1, and as tumor grade increased, the mRAN expression of CBX7 tended to be lower (**G**). *p<0.05, **p<0.01, ***p<0.001.

After mRNA expression and protein expression were found to be over-expressed in HCC patients, we next analyzed the relationship between mRNA expression of different CBXs family members with clinico-pathological parameters of HCC patients by UALCAN (http://ualcan.path.uab.edu), including patients’ individual cancer stages and tumor grades. As was shown in Figure 4, mRNA expressions of 8 CBXs family members were remarkably correlated with patients’ individual cancer stages, and patients who were in more advanced cancer stages tended to express higher mRNA expression of CBXs. The highest mRNA expressions of CBX4/8 were found in stage 4 (Figure 4D, H), while the highest mRNA expressions of CBX1/2/3/5/6/7 were found in stage 3 (Figure 4A-C, E-G). The reason why mRNA expressions of CBX1/2/3/5/6/7 in stage 3 seemed to be higher than that in stage 4 may be due to the small sample size (only 6 HCC patients were at stage 4). Similarly, as was shown in Figure 5, mRNA expressions of 8 CBXs family members were significantly related to tumor grades, and, as tumor grade increased, the mRNA expression of CBXs tended to be higher. The highest mRNA expressions of CBX1/3/4/5/6/8 were found in tumor grade 4 (Figure 5A, C-F, H), while the highest mRNA expression of CBX2 was found in grade 3 (Figure 5B). However, the highest mRNA expression of CBX7 was found in grade 1, and as tumor grade increased, the mRAN expression of CBX7 tended to be lower (Figure 5G). In short, the results above suggested that mRNA expressions of 8 CBXs family members were significantly associated with clinicopathological parameters in HCC patients.

### Prognostic value of mRNA expression of CBXs in liver cancer patients

**Figure 6:**
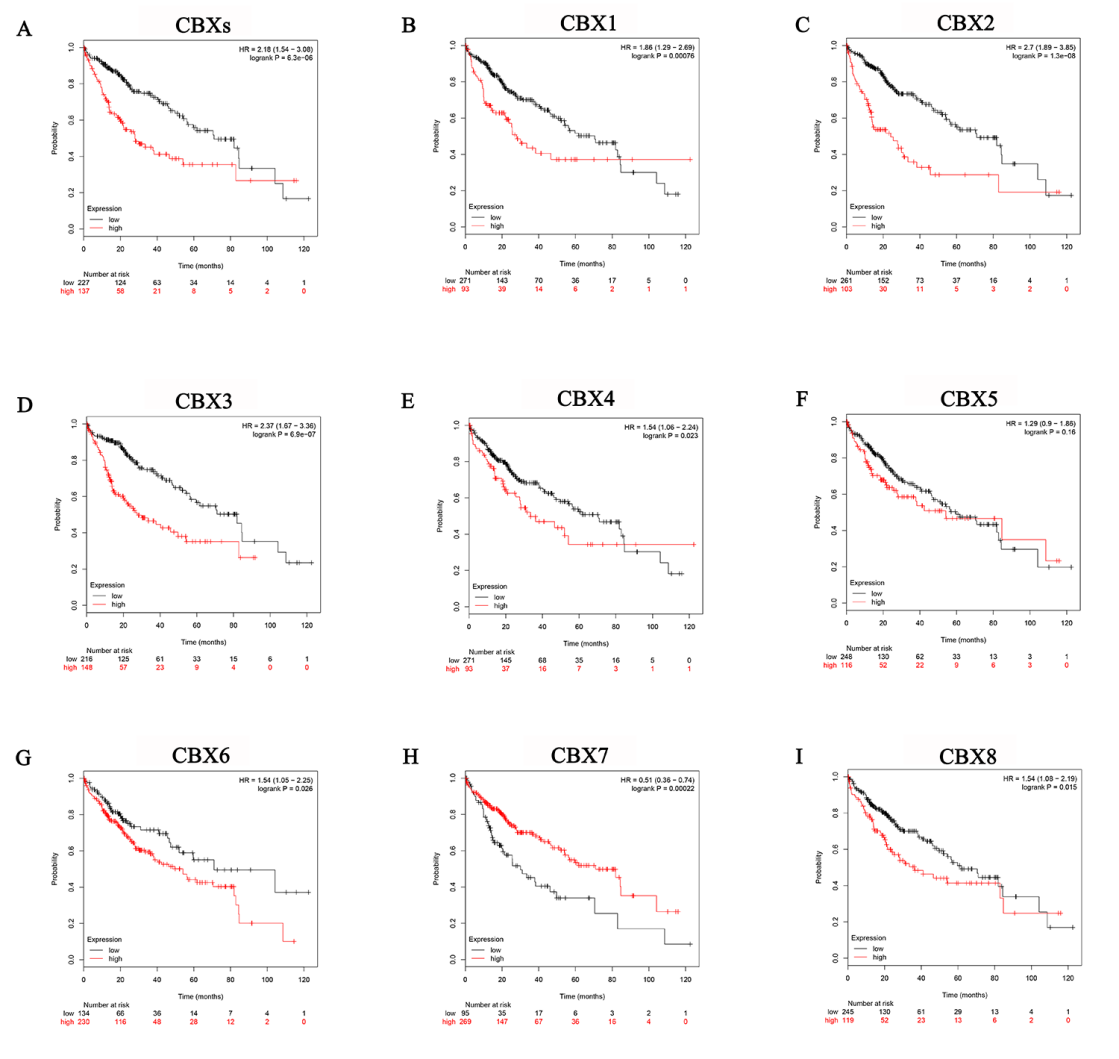
**Prognostic value of mRNA expression of distinct CBXs family members in liver cancer patients (Kaplan-Meier Plotter).** Generally, higher combinatory mRNA expressions of all 8 CBXs family members were associated with poorer OS in liver cancers patients (**A**). Specifically, higher mRNA expressions of CBX1/2/3/4/6/8 were significantly associated with shorter OS of liver cancers patients (**B-E, G, I**), while higher mRNA expression of CBX7 was significantly related to favorable OS of liver cancer patients (**H**). However, CBX5 mRNA expression showed no correlation with prognosis in liver cancer patients (**F**).

Further, we used Kaplan-Meier plotter (http://kmplot.com/analysis/) to analyze the prognostic values of the mRNA expression of CBXs in liver cancer patients. As was shown in Figure 6, mRNA expressions of most of the CBXs family members were significantly associated with liver cancer patients’ prognosis. First, the relationship between combinatory mRNA expressions of all 8 CBXs family members and prognosis of liver cancer patients was analyzed (Figure 6A). Our results showed that higher combinatory mRNA expressions of all 8 CBXs family members was associated with poorer OS in liver cancers patients (HR=2.18, 95% CI: 1.54-3.08, and p=6.3E-06). Next, the association between mRNA expression of distinct CBXs family members and prognosis of liver cancer patients were further analyzed. As were shown in Figure 6B-6E, Figure 6G and Figure 6I, higher mRNA expression of CBX1 (HR=2.11, 95% CI: 1.45-3.07, and p=7.2e-05), CBX2 (HR=2.7, 95% CI: 1.89-3.85, and p=1.3E-05), CBX3 (HR=2.37, 95% CI: 1.67-3.36, and p=6.9E-07), CBX4 (HR=1.54, 95% CI: 1.06-2.24, and p=0.023), CBX6 (HR=1.54, 95% CI: 1.05-2.25, and p=0.026), CBX8 (HR=1.54, 95% CI: 1.08-2.19, and p=0.015) were significantly associated with shorter OS of liver cancers patients, while higher mRNA expression of CBX7 was significantly related to favorable OS of liver cancer patients (HR=0.51, 95% CI: 0.36-0.74, and p=0.00022) (Figure 6H). However, CBX5 mRNA expression showed no correlation with prognosis of liver cancer patients (HR=1.29, 95% CI: 0.9-1.86, and p=0.016) (Figure 6F). These results indicated that mRNA expressions of CBX1/2/3/4/6/7/8 were significantly associated with liver cancer patients’ prognosis and they may be exploited as useful biomarkers for prediction of liver cancer patients’ survival.

### Independent prognostic value of mRNA expression of CBXs in terms of OS in liver cancer patients

After mRNA expressions of CBX1/2/3/4/6/7/8 were found to be significantly associated with liver cancer patients’ prognosis, we then tried to assess the independent prognostic value of mRNA expression of CBXs in terms of OS in liver cancer patients. We downloaded clinical data (Supplementary Table 1) and mRNA expression of CBXs of 364 HCC patients of TCGA database from the Firebrowse website (http://firebrowse.org/api-docs/) for Cox survival regression analysis. In univariate analysis, we found that high pathologic stage (HR=1.586, 95% CI: 1.304-1.929, and p<0.001), high mRNA expressions of CBX1 (HR=1.560, 95% CI: 1.192-2.040, and p=0.001), CBX2 (HR= 1.337, 95% CI: 1.196-1.494, and p<0.001), CBX3 (HR= 1.787, 95% CI: 1.247-2.561, and p=0.002), CBX6 (HR= 1.150, 95% CI: 1.025-1.290, and p=0.017), CBX8 (HR= 1.325, 95% CI: 1.056-1.663, and p=0.015) and low mRNA expression of CBX7 (HR= 0.792, 95% CI: 0.645-0.971, and p=0.025) were related to shorter OS of liver cancer patients (Supplementary Table 2). Multivariate analysis showed that high mRNA expressions of CBX1 (HR= 1.534, 95% CI: 1.167-2.018, and p=0.002), CBX2 (HR= 1.349, 95% CI: 1.198-1.519, and p<0.001), CBX3 (HR= 1.691, 95% CI: 1.175-2.432, and p=0.005), CBX6 (HR= 1.124, 95% CI: 1.001-1.261, and p=0.048), CBX8 (HR= 1.300, 95% CI: 1.036-1.631, and p=0.023) were independently associated with significantly shorter OS of liver cancer patients (Supplementary Tables 3-10). These results showed that transcriptional expressions of CBX1/2/3/6/8 were independent prognostic factors for OS of liver cancer patients.

### Genetic mutations in CBXs and their associations with OS and disease-free survival (DFS) of HCC patients

**Figure 7:**
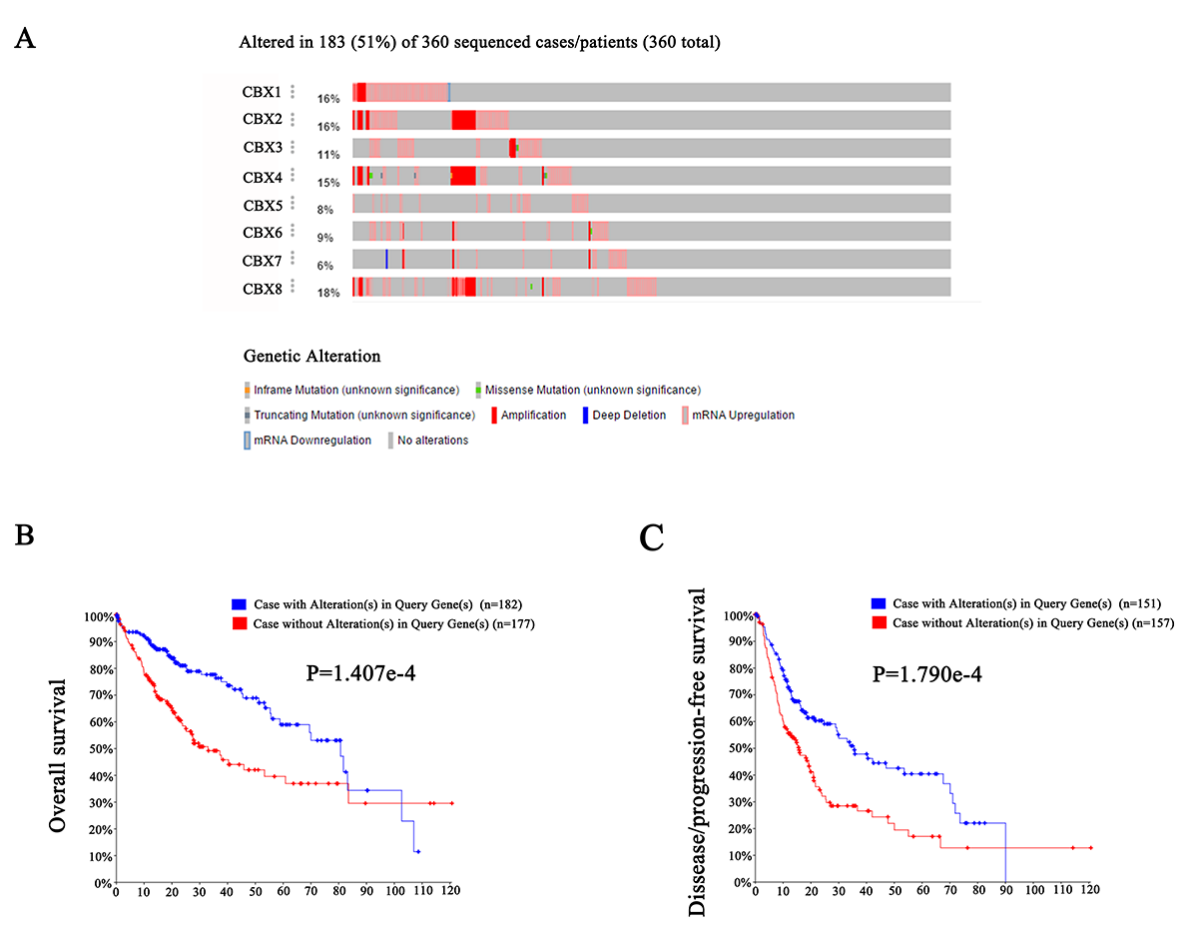
**Genetic mutations in CBXs and their association with OS and DFS of HCC patients (cBioPortal).** High mutation rate (51%) of CBXs was observed in HCC patients. CBX8, CBX1, CBX2 and CBX4 ranked the highest four genes of genetic alterations, and their mutation rates were 18%, 16%, 16% and 15%, respectively (**A**). Genetic alterations in CBXs were associated with shorter OS (**B**) and DFS (**C**) of HCC patients.

Next, we analyzed genetic alteration in CBXs and their associations with OS and DFS of HCC patients. As was shown in Figure 7A, high mutation rate of CBXs was observed in HCC patients. In the 360 sequenced HCC patients, genetic alteration was found in 183 HCC patients and the mutation rate was 51%. CBX8, CBX1, CBX2 and CBX4 ranked the highest four genes with genetic alterations, and their mutation rates were 18%, 16%, 16% and 15%, respectively. Furthermore, results from Kaplan-Meier plot and log-rank test showed that genetic alteration in CBXs was associated with shorter OS (Figure 7B, p=1.407E-4) and DFS (Figure 7C, p=1.790E-4) of HCC patients. These results implied that genetic alteration of CBXs could also significantly affect HCC patients’ prognosis.

### Predicted functions and pathways of the mutations in CBXs and their 50 frequently altered neighbor genes in HCC patients

**Figure 8:**
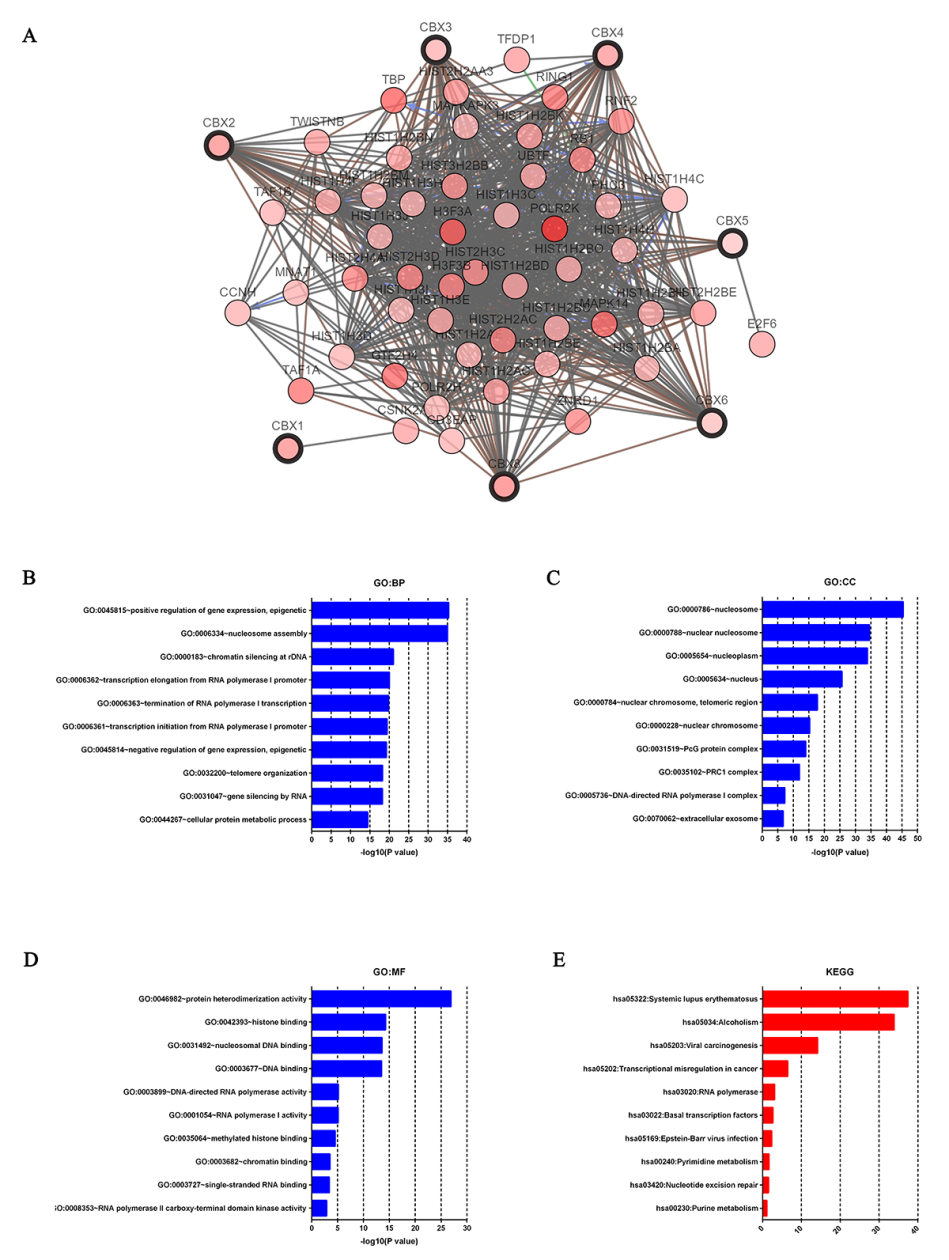
**Predicted functions and pathways of the mutations in CBXs and their 50 frequently altered neighbor genes in HCC patients (c-BioPortal and DAVID).** Network of CBXs mutations and their 50 frequently altered neighbor genes was constructed. DNA repair and DNA replication related genes including *HIST1H3A, HIST1H2AB, H3F3A, HIST2H2AA3, HIST2H3C* and *HIST3H2BB* were significantly related to CBXs mutations (**A**). GO functional enrichment analysis predicted three main functions of CBXs mutations and their 50 frequently altered neighbor genes, including biological process, cellular components and molecular functions (**B-D**). KEGG pathway analysis on CBXs and their 50 most frequently altered neighbor genes was shown at figure **E**.

After analyzing the genetic alterations in CBXs and their prognostic value in HCC patients, we further analyzed 50 neighbor genes which were significantly associated with CBXs mutations and constructed an integrated network by cBioPortal (www.cbioportal.org). As was shown at Figure 8A, the DNA repair and DNA replication related genes including *HIST1H3A, HIST1H2AB, H3F3A, HIST2H2AA3, HIST2H3C* and *HIST3H2BB* were significantly related to CBXs mutations. Moreover, functions of CBXs and their 50 frequently altered neighbor genes were analyzed by GO and KEGG in DAVID (https://david.ncifcrf.gov/summary.jsp). As were shown in Figure 8B-8D, biological processes such as GO: 0045815 (positive regulation of gene expression, epigenetic), GO: 0006334 (nucleosome assembly), GO: 0000183 (chromatin silencing at rDNA), GO: 0006362 (transcription elongation from RNA polymerase I promoter) and GO: 0006363 (termination of RNA polymerase I transcription) were remarkably regulated by the CBXs mutations in HCC (Figure 8B). Cellular components, including GO: 0000786 (nucleosome), GO: 0000788 (nuclear nucleosome), GO: 0005654 (nucleoplasm), GO: 0005634 (nucleus) and GO: 0000784 (nuclear chromosome, telomeric region) were significantly associated with the CBXs alterations (Figure 8C). In addition, CBXs mutations also prominently affected the molecular functions, such as GO: 0046982 (protein heterodimerization activity), GO: 0042393 (histone binding), GO: 0031492 (nucleosomal DNA binding), GO: 0003677 (DNA binding) and GO: 0003899 (DNA-directed RNA polymerase activity) (Figure 8D). In KEGG analysis, 6 pathways including has: 05322 (systemic lupus erythematosus), has: 05034 (alcoholism), has: 05203(viral carcinogenesis), has: 05202 (transcriptional misregulation in cancer) and has: 03020 (RNA polymerase) were associated with the functions of CBXs mutations in HCC (Figure 8E).

## DISCUSSION

In addition to cancer genetics, aberrant epigenetic regulation has also been found to take part in the development and progression and of HCC [4]. Being important components of epigenetic regulation complexes, CBXs family proteins are implicated in the development of multiple cancers, including HCC [9]. Despite some members of CBXs have been confirmed to play key roles in HCC, distinct roles of CBXs family members in HCC remained to be elucidated. In this study, the expression, mutation and prognostic values of different CBXs family members in HCC were analyzed.

Results from our study showed that over-expressions of mRNA and protein were found in all the 8 CBXs family members, and mRNA expression of CBXs was significantly associated with patients’ individual cancer stages and tumor grades in HCC patients. Besides, higher mRNA expressions of CBX1/2/3/6/8 were significantly associated with shorter OS in liver cancers patients, while higher mRNA expression of CBX7 was significantly related to favorable OS in liver cancer patients. Multivariate analysis showed that high mRNA expressions of CBX1/2/3/6/8 were independent prognostic factors for shorter OS of liver cancer patients. Moreover, high mutation rate (51%) of CBXs was observed in HCC patients and the genetic alteration in CBXs was associated with shorter OS and DFS in HCC patients. Finally, functions and pathways of the mutations in CBXs and their 50 frequently altered neighbor genes in HCC patients were analyzed and our results showed that the DNA repair and DNA replication related genes including *HIST1H3A, HIST1H2AB,* and *HIST3H2BB* were significantly related to CBXs mutations. Biological processes such as GO: 0045815 (positive regulation of gene expression, epigenetic), cellular components such as GO: 0000786 (nucleosome), molecular functions such as GO: 0046982 (protein heterodimerization activity), pathways such as, has: 05202 (transcriptional misregulation in cancer) were remarkably regulated by the CBXs mutations in HCC.

Over-expression of CBX1 had been found in castration-resistant prostate cancer (CRPC) and breast cancers (BC) [18,19]. In prostate cancer (PCa) tissues, higher CBX1 expression correlated with Gleason score and tri-methylation levels of histone H3K9. Inhibition of CBX1 suppressed the growth of androgen/androgen receptor-expressing PCa cells via inducing cell-cycle arrest at the G1 phase [18]. Similarly, over-expression of CBX1 was also found to be related with poorly differentiated breast tumors and poorer prognosis of BC patients [19]. Recently, the expression of CBX1 was found to be noticeably over-expressed in HCC tissues and cell lines. High CBX1 expression was associated with larger tumor size, poor tumor differentiation, tumor vascular invasion and unfavorable OS and DFS in HCC cases. In vitro study demonstrated that CBX1 over-expression promoted HCC cells proliferation and migration by interacting with transcription factor HMGA2 to activate the Wnt/β-Catenin signaling pathway, whereas knockdown of CBX1 or suppression of β-Catenin markedly decreased CBX1-mediated cell growth [20]. In our present study, significantly higher mRNA and protein expressions of CBX1 were found in HCC tissues compared to normal tissues, and that the mRNA expression of CBX1 was significantly associated with patients’ individual cancer stages and tumor grades which was in accordance with previous studies. Besides, higher mRNA expression of CBX1 was also significantly related with shorter OS of liver cancers patients and was an independent prognostic factor for shorter OS of liver cancer patients, indicating CBX1 took part in the tumorigenesis of HCC.

Recently, Clermont et al. conducted a genotranscriptomic meta-analysis of CBX2 in human cancers and found that CBX2 mRNA expression in many cancers was higher than that in normal tissues, which was independent of CDKN2A/B silence. Besides, over-expression and amplification of CBX2 were significantly related with metastatic progression and shorter OS in many cancer types, particularly in BC patients [21]. Mechanistically, Di Costanzo et al. found that CBX2 was over-expressed in leukemic cells and knockdown of CBX2 suppressed the tumorigenic properties and self-renewal capability of leukemic cells [22]. Clermont et al. also observed that CBX2 depletion abrogated cell viability and induced caspase3-mediated apoptosis in metastatic PCa cell lines [23]. In our study, significantly higher mRNA and protein expression of CBX2 were found in HCC tissues, and mRNA expression of CBX2 was remarkably correlated with patients’ individual cancer stages and tumor grades, which were similar to the findings of Clermont’ studies [21]. Moreover, higher mRNA expression of CBX2 was also significantly related with poorer OS of liver cancers patients and was an independent prognostic factor for shorter OS of liver cancer patients, indicating an oncogenic role of CBX2 in the HCC.

Significant up-regulation of CBX3 had been found in a variety of cancers, including lung adenocarcinoma (LUAD) and non-small cell lung cancer (nSCLC), tongue squamous cell carcinoma (TSCC), and colorectal cancer [24–27]. Studies from Liu et at showed that CBX3 was over-expressed in human colorectal cancer and it promoted cell proliferation of colorectal cancer cell lines by directly regulating CDKN1A in a manner associated with methylation of histone H3K9 on its promoter. Moreover, miR-30a could target CBX3 in vitro and in vivo to specifically inhibit the growth of colorectal cancer in mouse xenograft models [26]. Alam et al. also revealed that CBX3 was one of the most frequently over-expressed and amplified histone reader proteins in human LUAD and that high CBX3 mRNA level was associated with poor prognosis of LUAD patients. CBX3 promoted the proliferation, colony formation, and migration of LUAD cells by directly repressing NCOR2 and ZBTB7A. In vivo depletion of CBX3 suppressed K-RasG12D-driven LUAD and increased survival of mice bearing K-RasG12D-induced LUAD [25]. In the present study, significantly higher mRNA and protein expressions of CBX3 were found in HCC tissues, and mRNA expression of CBX3 was dramatically associated with patients’ individual cancer stages and tumor grades which was consistent with the studies above. Higher mRNA expression of CBX3 was also significantly related with unfavorable OS of liver cancers patients and was an independent prognostic factor for shorter OS of liver cancer patients, which indicated that CBX3 took part in the tumorigenesis of HCC.

CBX4 is the most well studied member of the CBXs family in HCC. Studies carried out by Jiao et al. and Wang et al. showed that CBX4 was over-expressed in clinical tissues and multiple HCC cell lines. Higher expression ofCBX4 was associated with clinical parameters including α-fetoprotein level, tumor size, pathologic differentiation, shorter OS and RFS [14,28]. Mechanistically, Li et al. found that CBX4 over-expression promoted tumor progression by increasing VEGF production and angiogenesis under hypoxia in subcutaneously and orthotopically transplanted HCC mice, while endogenous knockdown of CBX4 eliminated the oncogenic effect of CBX4 [29]. Moreover, Zhang et al. had revealed that miR-195 could significantly inhibit the proliferative, invasive and migratory capacities of HepG2 cells and HCC growth in vivo experiments by down-regulation of CBX4 [30]. Similar tumorigenic effect of CBX4 in HCC was also found in our present study. Our results showed that higher mRNA and protein expressions of CBX4 were found in HCC tissues, and that mRNA expression of CBX4 was significantly related with patients’ individual cancer stages and tumor grades.

Similar to CBX3, over-expression of CBX5 was found in many kinds of malignancies, such as pancreatic cancers, breast cancers and lung cancers [31]. In BC patients, higher CBX5 expression was correlated with decreased survival and increased occurrence of metastasis over time. Using CBX5 expression to predict disease outcome was a better prognostic biomarker than standard prognostic ones. Besides, down-regulation of CBX5 resulted in mitotic defects of breast cancer cell lines Hs578T [32]. In lung cancer patients, mRNA expression of *CBX5* was significantly higher in the tumor samples as well as in the metastatic lesions and was associated with worse OS. Moreover, knockdown of CBX5 significantly inhibited capabilities of sphere and colony formation, migration of CD133^+^-tumor stem-like cells (TSLCs) in vitro and the tumorigenic engraftment, tumor growth rate, and metastatic tendency to lung caused by lung CD133^+^-TSLCs in vivo [33]. In our study, mRNA and protein expressions of CBX5 were found to be significantly higher in HCC tissues, and mRNA expression of CBX5 was significantly related with patients’ individual cancer stages and tumor grades. Despite patients with shorter OS tended to express higher mRNA expression of CBX5, the difference was not statistically significant. Further verifications are needed to discuss whether CBX5 plays an oncogenic role in HCC as other CBXs family members.

Frequent up-regulation of CBX6 had been found in HCC tissues and HCC cell lines and that CBX6 expression was significantly related with tumor sizes and multiple tumors. HCC patients with higher CBX6 expression had significantly shorter RFS and OS than those with lower CBX6 expression, and increased CBX6 expression was an independent unfavorable prognostic factor for HCC patients. Moreover, mechanistic study had shown that over-expression of CBX6 profoundly promoted HCC cell growth both in vitro and in vivo by regulating S100A9/NF-κB/MAPK pathway [34]. Similarly, in our study, higher mRNA expression of CBX6 was found in HCC tissues compared to normal tissues, and was significantly related with patients’ individual cancer stages, tumor grades. CBX6 was also significantly related with shorter OS of liver cancers patients and was an independent prognostic factor for shorter OS of liver cancer patients. All these results showed that CBX6 contributed to the development and progression of HCC and it may serve as a novel prognostic biomarker in HCC treatment.

Conflicting roles of CBX7 had been found in different kinds of human cancers [35]. On one hand, decreased expression of CBX7 had been found in most of the human malignant carcinomas, including bladder cancers, thyroid cancers, colorectal cancers, breast cancers and lung carcinomas, and in these cancers, down-regulation of CBX7 had been shown to correlate with cancer aggressiveness and poor prognosis, suggesting an oncosuppressor role of CBX7 in these cancers [36–40]. Mechanistic studies had found that CBX7 was able to counteract the oncogenic function of the HMGA proteins and to inhibit the expression of proliferation related and migration related genes, such as CCNE and SPP1 [35]. On the other hand, over-expression of CBX7 had also been found in some malignancies, such as prostate cancers and ovarian cancers [41,42]. Patients with over-expression of CBX7 exhibited reduced overall and progression-free survival rates compared to those expressing lower CBX7. Accordingly, inhibition of CBX7 decreased cell viability of ovarian carcinoma cell lines by promoting expression of TRAIL [42]. In regard to HCC, down-regulation of CBX7 had been found in HCC tissues and was associated with shorter OS of HCC patients [15]. Moreover, over-expression of miR-18a promoted cell proliferation and migration of HCC cell lines partly through decreasing CBX7 and depletion of CBX7 had the similar effects as miR-18a over-expression on HCC cell lines [43]. In our study, conflicting findings about the role of CBX7 in HCC were observed. On one hand, higher mRNA and protein expressions of CBX7 were found in HCC tissues, and mRNA expression of CBX7 was significantly related with patients’ individual cancer stages and tumor grades. However, on other hand, higher mRNA expression of CBX7 was correlated with better OS in liver cancers patients. Therefore, further studies are still required to assess the exact role of CBX7 in HCC.

Increased expression of CBX8 had been found in HCC tissues and was associated with poor prognosis of HCC patients [44]. Functional study had showed that over-expression of CBX8 promoted tumor growth and metastasis by increasing EGR1 and miR-365-3p to stimulate the AKT/β-catenin pathway, while CBX8 inhibition suppressed these effects [45]. Likewise, in the present study, significantly higher mRNA and protein expression of CBX8 were also found in HCC tissues, mRNA expression of CBX8 was remarkably correlated with patients’ individual cancer stages and tumor grades. Accordingly, higher mRNA expression of CBX8 was also significantly related with shorter OS of liver cancers patients and was an independent prognostic factor for shorter OS of liver cancer patients. Together with other findings discussed above, our results suggested that CBX8 played an oncogenic role in HCC.

There were some limitations in our study. First, although high mRNA expressions of CBX1/2/3/6/8 were independent prognostic factors for shorter OS of liver cancer patients, all the data analyzed in our study was retrieved from the online databases, further studies consist of larger sample sizes are required to validate our findings and to explore the clinical application of the CBXs members in the treatment of HCC. Second, we did not assess the potential diagnostic and therapeutic roles of CBXs in HCC, so future studies are needed to explore whether CBXs could be exploited as diagnostic markers or as therapeutic targets. Finally, we did not explore the potential mechanisms of distinct CBXs in HCC. Future studies worth to investigate the detailed mechanism between distinct CBXs and HCC.

In conclusion, our results showed that over expressions of 8 CBXs members were found to be significantly associated with clinical cancer stages and pathological tumor grades in HCC patients. Besides, higher mRNA expressions of CBX1/2/3/6/8 were found to be significantly associated with OS in HCC patients, while higher mRNA expression of CBX7 was associated with favorable OS. Multivariate analysis also showed that high mRNA expressions of CBX1/2/3/6/8 were independent prognostic factors for shorter OS of liver cancer patients. Moreover, high mutation rate of CBXs (51%) was also observed in HCC patients, and genetic alteration in CBXs was associated with shorter OS and DFS in HCC patients. These results indicated that CBX1/2/3/6/8 could be prognostic biomarkers for survivals of HCC patients.

## MATERIALS AND METHODS

### Ethics statement

Our study protocol was approved by the Ethics Committee of the Third Affiliated Hospital of Sun Yat-sen University. As all the data were retrieved from the online databases, so it could be confirmed that all written informed consent had already been obtained.

### ONCOMINE database

ONCOMINE database (www.oncomine.org) is an integrated online cancer microarray database for DNA or RNA sequences analysis, which aims to facilitate discovery from the gene-wide expression analyses [46]. In our study, transcriptional expressions of 8 different CBXs members between different cancer tissues and their corresponding adjacent normal control samples were got from ONCOMINE database. Difference of transcriptional expression was compared by students’ t-test. Cut-off of *p* value and fold change were as following: *p* value: 0.01, fold change: 1.5, gene rank: 10%, data type: mRNA.

### UALCAN

UALCAN (http://ualcan.path.uab.edu) is an interactive web resource based on level 3 RNA-seq and clinical data of 31 cancer types from TCGA database. It can be used to analyze relative transcriptional expression of potential genes of interest between tumor and normal samples and association of the transcriptional expression with relative clinicopathologic parameters [47]. In this study, UALCAN was used to analyze the mRNA expressions of 8 CBXs family members in primary HCC tissues and their association with clinicopathologic parameters. Difference of transcriptional expression was compared by students’ t test and *p* <0.01 was considered as statically significant.

### Human Protein Atlas

The Human Protein Atlas (https://www.proteinatlas.org) is a website that contains immunohistochemistry-based expression data for near 20 highly common kinds of cancers and each tumor type includes 12 individual tumors [48]. Users can identify tumor-type specific proteins expression patterns that are differentially expressed in a given tumors of type. In this study, direct comparison of protein expression of different CBXs family members between human normal and HCC tissues was performed by immunohistochemistry image.

### Kaplan-Meier plotter

The prognostic value of mRNA expression of distinct CBXs in liver cancers was analyzed by using Kaplan-Meier plotter (http://kmplot.com/analysis/),in which information about association of gene expression with survival of patients of liver cancer, breast cancer, ovarian cancer, lung cancer and gastric cancer could be easily access to [49–52]. In Kaplan-Meier plotter, cancer patients were divided into high and low expression group based on median values of mRNA expression and validated by K-M survival curves. Information about the number-at-risk cases, median values of mRNA expression levels, HRs, 95% CIs and *p*-values can be found at the K-M plotter webpage. Statically significant difference was considered when a *p* value < 0.05.

### Cancer Genome Atlas (TCGA) database

TCGA is a comprehensive and coordinated project designed to improve diagnosis methods, treatment standards, and ultimately to prevent cancer. Information about sequencing and pathological data of more than 30 kinds of human tumors can be analyzed in TCGA [53]. In our analysis, clinicopathological parameters of 377 HCC patients and mRNA expression of CBXs of 371 HCC patients were downloaded from the Firebrowse website (http://firebrowse.org/api-docs/). 7 of 377 HCC patients were excluded because of the absence of follow-up data. Finally, 364 HCC patients subjected to mRNA expression of CBXs were included in our analysis. Clinical data, including gender, age, weight, PLT, albumin, creatinine, prothrombin time, total bilirubin, AFP, Child-Pugh stage, adjacent tissue inflammation, cirrhosis, histologic grade and pathologic stage were summarized in Supplementary Table 1.

### cBioPortal

cBioPortal (www.cbioportal.org) is an online open-access website resource for exploring, visualizing, and analyzing multidimensional cancer genomics data [54]. In this study, we analyzed the genomic profiles of 8 CBXs family members, which contained mutations, putative copy-number alterations from GISTIC and mRNA Expression z-Scores (RNASeq V2 RSEM) with a z-score threshold ±1.8. Genetic mutations in CBXs and their association with OS and DFS of HCC patients were displayed as Kaplan-Meier plots and log-rank test was performed to identify the significance of the difference between the survival curves, and when a p value <0.05, the difference was considered statically significant.

### Gene Ontology (GO) and Kyoto Encyclopedia of Genes and Genomes (KEGG) analysis

Functions of CBXs mutations and 50 genes significantly associated with CBX mutations were analyzed by GO and KEGG in the Database for Annotation, Visualization, and Integrated Discovery (*DAVID*) (https://david.ncifcrf.gov/summary.jsp). GO enrichment analysis can predict the functional roles of CBXs mutations and 50 genes significantly associated with CBX mutations on the basis of three aspects, including biological processes(BP), cellular components (CC), and molecular functions (MF), while KEGG analysis can define the pathways related to the CBXs mutations and 50 frequently neighbor genes associated with CBXs mutations.

### Statistical methods

Cox regression analysis was used to evaluate the association of mRNA expression of CBXs with patient survival by SPSS software version 20.0. First, missing covariates were imputed with methods similar to the methods of White [55]. Then, effect of clinical parameters and mRNA expression of CBXs on survival of HCC patients was evaluated by univariate Cox regression, followed by a filter which retained those with p ≤ 0.1 for subsequent analysis. Finally, association of mRNA expression of CBXs with patient survival was further analyzed with multivariate Cox regression, which adjusted for other parameters (e.g. Child-Pugh stage, histologic grade) similar to the methods of Hou [56]. P < 0.05 was considered statistically significant.

## SUPPLEMENTARY MATERIAL

Supplementary Table 1

Supplementary Table 2

Supplementary Table 3-10
