# Supplementary material for: Transcriptional expressions of Chromobox 1/2/3/6/8 as independent indicators for survivals in hepatocellular carcinoma patients
Source: Aging (Albany NY). 2018 Nov 27;10(11):3450–73. doi: 10.18632/aging.101658 (PMC6286817; doi:10.18632/aging.101658)
Supplement: Supplementary Table 1 [file aging-10-101658-s001.docx]

**Supplementary Table 1. Basic characteristics of 364 HCC patients.**

| **Variables** | **HCC patients（N=364）** |
| --- | --- |
|  |  |
| Gender( Male/female) | 246/118 |
| Age(years, Mean±SD) | 59.67±13.37 |
| Weight（kg, Median） | 69(40-172) |
| PLT (10e9/L, Median) | 211(4-499000) |
| Albumin (g/L, Median) | 4(0.2-5200) |
| Creatinine(mg/dl, Median) | 0.9(0.4-124) |
| PLT (10e9/L, Median) | 211(4-499000) |
| PT (s, Median) | 1.1(0.8-36.4) |
| TB (μmol/L, Median) | 1.2(0.2-21) |
| AFP (ng/ml, Median) | 15(1-2035400) |
| Childpugh stage |  |
| A | N=216 |
| B | N=21 |
| C | N=1 |
| Adjacent tissue inflammation |  |
| Non | N=117 |
| Mild | N=97 |
| Severe | N=17 |
| Cirrhosis |  |
| Non-cirrhosis | N=74 |
| Cirrhosis | N=134 |
| Histologic grade |  |
| 1 | N=55 |
| 2 | N=174 |
| 3 | N=118 |
| 4 | N=12 |
| Pathologic stage |  |
| 1 | N=170 |
| 2 | N=83 |
| 3 | N=83 |
| 4 | N=4 |

HCC:hepatocellular carcinoma, SD:standard deviation, PT: prothrombin time, TB:total bilirubin
