# Supplementary material for: Transcriptional expressions of Chromobox 1/2/3/6/8 as independent indicators for survivals in hepatocellular carcinoma patients
Source: Aging (Albany NY). 2018 Nov 27;10(11):3450–73. doi: 10.18632/aging.101658 (PMC6286817; doi:10.18632/aging.101658)
Supplement: Supplementary Table 2 [file aging-10-101658-s002.docx]

**Supplementary Table 2. Univariate analysis of overall survival in 364 HCC specimens.**

| **Variables** | **Univariate analysis** | | |
| --- | --- | --- | --- |
|  | **Hazard ratio** | **95% CI** | **P value** |
| Gender | 0.816 | 0.573-1.163 | 0.260 |
| Age(years) | 1.012 | 0.999-1.026 | 0.078 |
| Weight（kg） | 0.993 | 0.984-1.003 | 0.189 |
| Adjacent tissue inflammation | 1.119 | 0.819-1.528 | 0.481 |
| Albumin (g/L) | 1.000 | 0.998-1.001 | 0.629 |
| Childpugh stage | 1.408 | 0.822-2.410 | 0.213 |
| Creatinine | 1.002 | 0.987-1.017 | 0.786 |
| AFP (ng/ml) | 1.000 | 1.000-1.000 | 0.335 |
| PLT (10e9/L) | 1.000 | 1.000-1.000 | 0.729 |
| PT (s) | 1.010 | 0.976-1.046 | 0.569 |
| TB (μmol/L) | 0.966 | 0.863-1.082 | 0.554 |
| Cirrhosis | 0.864 | 0.560-1.322 | 0.508 |
| Histologic grade | 1.122 | 0.889-1.416 | 0.332 |
| Pathologic stage | 1.586 | 1.304-1.929 | 0.000* |
| CBX1 | 1.560 | 1.192-2.040 | 0.001* |
| CBX2 | 1.337 | 1.196-1.494 | 0.000* |
| CBX3 | 1.787 | 1.247-2.561 | 0.002* |
| CBX4 | 1.179 | 0.932-1.490 | 0.169 |
| CBX5 | 1.050 | 0.851-1.294 | 0.650 |
| CBX6 | 1.150 | 1.025-1.290 | 0.017* |
| CBX7 | 0.792 | 0.645-0.971 | 0.025* |
| CBX8 | 1.325 | 1.056-1.663 | 0.015* |

HCC:hepatocellular carcinoma, PT: prothrombin time, TB:total bilirubin
