# Supplementary material for: Transcriptional expressions of Chromobox 1/2/3/6/8 as independent indicators for survivals in hepatocellular carcinoma patients
Source: Aging (Albany NY). 2018 Nov 27;10(11):3450–73. doi: 10.18632/aging.101658 (PMC6286817; doi:10.18632/aging.101658)
Supplement: Supplementary Table 3-10 [file aging-10-101658-s003.docx]

**Supplementary Table 3. Multivariate analysis of overall survival in 364 HCC specimens.**

| **Variables** | **Multivariate analysis** | | |
| --- | --- | --- | --- |
|  | **Hazard ratio** | **95% CI** | **P value** |
| Age(years) | 1.016 | 1.002-1.031 | 0.023* |
| Adjacent tissue inflammation |  |  |  |
| Childpugh stage |  |  |  |
| AFP (ng/ml) |  |  |  |
| Cirrhosis |  |  |  |
| Histologic grade |  |  |  |
| Pathologic stage | 1.541 | 1.260-1.884 | 0.000* |
| CBX1 | 1.534 | 1.167-2.018 | 0.002* |

**Supplementary Table 4. Multivariate analysis of overall survival in 364 HCC specimens.**

| **Variables** | **Multivariate analysis** | | |
| --- | --- | --- | --- |
|  | **Hazard ratio** | **95% CI** | **P value** |
| Age(years) | 1.014 | 1.000-1.029 | 0.048* |
| Adjacent tissue inflammation |  |  |  |
| Childpugh stage | 1.768 | 1.004-3.113 | 0.048* |
| AFP (ng/ml) | 1.000 | 1.000-1.000 | 0.244 |
| Cirrhosis |  |  |  |
| Histologic grade |  |  |  |
| Pathologic stage | 1.456 | 1.192-1.777 | 0.000* |
| CBX2 | 1.349 | 1.198-1.519 | 0.000* |

**Supplementary Table 5. Multivariate analysis of overall survival in 364 HCC specimens.**

| **Variables** | **Multivariate analysis** | | |
| --- | --- | --- | --- |
|  | **Hazard ratio** | **95% CI** | **P value** |
| Age(years) | 1.016 | 1.002-1.030 | 0.022* |
| Adjacent tissue inflammation |  |  |  |
| Childpugh stage | 1.701 | 0.949-3.05 | 0.074 |
| AFP (ng/ml) | 1.000 | 1.000-1.000 | 0.407 |
| Cirrhosis |  |  |  |
| Histologic grade |  |  |  |
| Pathologic stage | 1.512 | 1.236-1.850 | 0.000* |
| CBX3 | 1.691 | 1.175-2.432 | 0.005* |

**Supplementary Table 6. Multivariate analysis of overall survival in 364 HCC specimens.**

| **Variables** | **Multivariate analysis** | | |
| --- | --- | --- | --- |
|  | **Hazard ratio** | **95% CI** | **P value** |
| Age(years) | 1.012 | 0.999-1.026 | 0.074 |
| Adjacent tissue inflammation |  |  |  |
| Childpugh stage |  |  |  |
| AFP (ng/ml) |  |  |  |
| Cirrhosis |  |  |  |
| Histologic grade |  |  |  |
| Pathologic stage | 1.592 | 1.307-1.939 | 0.000* |
| CBX4 |  |  |  |

**Supplementary Table 7. Multivariate analysis of overall survival in 364 HCC specimens.**

| **Variables** | **Multivariate analysis** | | |
| --- | --- | --- | --- |
|  | **Hazard ratio** | **95% CI** | **P value** |
| Age(years) | 1.012 | 0.999-1.026 | 0.074 |
| Adjacent tissue inflammation |  |  |  |
| Childpugh stage |  |  |  |
| AFP (ng/ml) |  |  |  |
| Cirrhosis |  |  |  |
| Histologic grade |  |  |  |
| Pathologic stage | 1.592 | 1.307-1.939 | 0.000* |
| CBX5 |  |  |  |

**Supplementary Table 8. Multivariate analysis of overall survival in 364 HCC specimens.**

| **Variables** | **Multivariate analysis** | | |
| --- | --- | --- | --- |
|  | **Hazard ratio** | **95% CI** | **P value** |
| Age(years) | 1.012 | 0.999-1.026 | 0.069 |
| Adjacent tissue inflammation |  |  |  |
| Childpugh stage |  |  |  |
| AFP (ng/ml) |  |  |  |
| Cirrhosis |  |  |  |
| Histologic grade |  |  |  |
| Pathologic stage | 1.567 | 1.284-1.912 | 0.000* |
| CBX6 | 1.124 | 1.001-1.261 | 0.048* |

**Supplementary Table 9. Multivariate analysis of overall survival in 364 HCC specimens.**

| **Variables** | **Multivariate analysis** | | |
| --- | --- | --- | --- |
|  | **Hazard ratio** | **95% CI** | **P value** |
| Age(years) | 1.014 | 1.002-1.028 | 0.042* |
| Adjacent tissue inflammation |  |  |  |
| Childpugh stage |  |  |  |
| AFP (ng/ml) |  |  |  |
| Cirrhosis |  |  |  |
| Histologic grade |  |  |  |
| Pathologic stage | 1.542 | 1.262-1.885 | 0.000* |
| CBX7 | 0.838 | 0.692-1.015 | 0.071 |

**Supplementary Table 10. Multivariate analysis of overall survival in 364 HCC specimens.**

| **Variables** | **Multivariate analysis** | | |
| --- | --- | --- | --- |
|  | **Hazard ratio** | **95% CI** | **P value** |
| Age(years) | 1.012 | 0.998-1.026 | 0.092 |
| Adjacent tissue inflammation |  |  |  |
| Childpugh stage |  |  |  |
| AFP (ng/ml) | 1.000 | 1.000-1.000 | 0.372 |
| Cirrhosis |  |  |  |
| Histologic grade |  |  |  |
| Pathologic stage | 1.563 | 1.282-1.906 | 0.000* |
| CBX8 | 1.300 | 1.036-1.631 | 0.023* |
